# Elements of Physiology, Including Physiological Anatomy, for the Use of the Medical Student

**Published:** 1846-09

**Authors:** 


ART. IV.—*Elements of Physiology, including Physiological Anatomy, for the use of the Medical Student; by* William B. Carpenter, M. D., F. R. S. etc., *with one hundred and eighty illustrations; 8vo. pp.* 559, *Lea & Blanchard, Philadelphia.*

This is a new work by Dr. Carpenter, republished by Lea and Blanchard with their usual promptitude, and, we may add, in that style of typographical excellence which characterises the publications emanating from their press.

The author, for many years a distinguished lecturer at Bristol, has recently been appointed to the chair of Physiology in the Royal Institution of Great Britain. In this country he is known more especially from his work on the *principles of human Physiology*, which here, as at home, has been received with almost universal commendation, and is recommended as a text book in most of our medical schools. He has also written a work on *General and Comparative Physiology*, which is soon to be issued from the press of Lea & Blanchard, and a popular treatise on *Vegetable Physiology*, which has already been reprinted by the same enterprising publishers. The present work is more elementary in its character than those which have preceded it, the object being, as stated in the preface, "to convey to the student as clear an idea as possible of the principles of the science, to point out the manner in which these principles should be applied, and to give an outline of the most important facts which indicate the nature of the various changes taking place in the living organism." The author apologises for appearing before the public so soon after his previous publications. In this he evinces modesty, which is always becoming, but we are sure that the public will not deem any apologetic explanation called for.

The expectations based upon his previous productions will lead the medical public to greet with warm interest any thing which Dr. Carpenter may write on the subject of Physiology, and these anticipations will be heightened by the perusal of the work now under consideration. To say that it is the best manual of Physiology now before the public, presenting the most satisfactory view of the general principles of the science in its present aspects and relations, would not do sufficient justice to the author; for much of this commendation would of necessity belong to the fact that it is the newest treatise on the subject. In a pursuit so eminently progressive, it follows, other things being equal, that the latest work is the best. New editions of old works in which an effort is made, by means of addenda, to incorporate fresh discoveries, and keep pace with the development of new views, must, always, fulfil but imperfectly the desired objects. It must happen that much which has been disproved by later investigations was so interwoven in the original texture, that when removed, the whole fabric is destroyed, or greatly mutilated. A work loses a great share of its originally fair proportions, by being curtailed and patched to suit the changes incident to the progressive march of science. It is much like endeavoring by successive additions to a small tenement, a great portion of which has already gone to decay, to erect a new and commodious edifice. Better far, pull down the old building, and commence anew upon a plan adapted to present resources and wishes.

Our readers will already be at no loss to surmise our estimation of the work whose title page is prefixed to this article. We are more than gratified, we are delighted with it. Writing fresh from its perusal, we find it somewhat difficult to repress a proneness to eulogise with more enthusiasm than would perhaps comport with grave maxims of critical prudence, when the subject is a scientific work. In truth it has been to us a rich treat, and we commend it in the warmest terms to our readers, having no apprehensions that their opinions of its merits will not fully justify our own. In ease and clearness of style, and lucid description, the work exhibits great improvement upon those which have preceded it by the same author. In these respects a marked difference will be observed. For example, in the "Principles of Human Physiology," the paramount object apparently was, to gather together all the latest accredited facts, without bestowing pains to present them in a form to be most readily and agreably comprehensible by the reader; but in the present work the chief aim, on the other hand, seems to have been to treat of the subject as simply and clearly as possible; and we are glad to see that in the elucidation of topics which, to those not practically conversant with microscopical investigations, are somewhat intricate and obscure, he divests them almost entirely of the multitude of new terms, which, however classically appropriate, are certainly far from being euphonous, and serve not a little to increase the labor and difficulty of becoming acquainted with the facts which late researches have developed. We allude now more particularly to the portions which treats of the philosophy of cells. Another excellent quality appertains to the work under notice: the author confines himself to what, in the present condition of the science, is chiefly interesting or important, without imposing on the reader a digest of all that has been heretofore received as true, but which is now obsolete. A history of old exploded doctrines is very well in its way, but it is a serious inconvenience for the reader who wishes with a reasonable economy of time and effort to inform himself of the facts which belong to the present condition of the science, to wade through all that has characterized its past annals, or to be under the necessity of discriminating important matter commingled with unprofitable or irrelevant discussions. Like all physiological works which have preceded it, and, we fear it must be added, like all which will follow, it doubtless contains many hypothetical speculations, which, in the progress of science, will be disproved. We do by no means aver that it is perfect. This would be, in other words, to affirm that the science had attained to perfection. What we mean to say is, that the writer has succeeded admirably in giving a plain graphical picture of the general principles of Physiology, as the science now exists, compressed within a compass which makes it readily available not only by the medical student, but by practitioners who have not leisure nor disposition to read elaborate publications and presented in a shape and style which enable the reader to apprehend, by an easy and pleasant process, the knowledge which the author wishes to convey.

A very considerable portion, nearly one fourth of the entire treatise, is devoted to a consideration of *cells*, their arrangement in constituting the solid structures and some of the fluids, and their offices in the various processes going on in the living organism. The cell-theory (for we must at present call it a theory,) is of recent date, but it now constitutes one of the most striking features of Physiology. Resulting from exploration of the wide field opened by the microscope, in which numerous able and industrious observers have been diligently occupied for the last few years, (and the coincidence of whose observations on important points is a strong argument in favor of their correctness,) it has expanded by the rapid accumulation of facts and inductions into an extensive science of itself. It seems to form an intermediate link between Anatomy and Physiology; and as a distinct department of investigation has received several titles—such as *Microscopical Anatomy*, *Physiological Anatomy*, and, significantly, but not very appropriately, *Trancendental Anatomy*. Assuming the theory to be true, histology or histogeny, which treats of the development of the tissues and organs, is necessarily a component of this new department of science. Its relations, however, to the active phenomena of the organism are so intricate and general, that it necessarily enters extensively into the consideration of physiological subjects. So recent are these investigations, and the new and curious principles to which they have given rise, that it would not be surprising if a large portion of the practical members of the profession have not, as yet, had time and opportunity to examine into their scope and character. In fact, it is only in the latest Physiological treatises [Muller, Carpenter, Wagner, Todd and Bowman, &c.] that they have received the attention due to their prominence and real importance. And even if some of these works have been consulted, it is quite probable that the dryness of minute detail, the absence of systematic arrangement, and the use of a new and barbarous nomenclature, have discouraged or disgusted many. Dr. Carpenter's work gives a more lucid, general account of our actual knowledge, conjoined with the theoretical views now recognized by some of the most eminent Physiologists, than any with which we are acquainted; and we advise those who have heretofore turned aside from the subject as one of the fantastical subtleties of the day, to forego a settled antipathy until they have given this work a perusal. As he has treated of the subject with as much condensation as is consistent with an elementary view, it is, of course, impracticable to subject this portion of his treatise to an analysis, without occupying as much space as does the author himself. In order, however, to enlist a little interest in the mind of the reader, preparatory to a perusal of the work, we would say that some of the leading features of the cell-theory are briefly as follows:

The primary and ultimate anatomical element or exemplar of all organized structures, vegetable and animal, is a *cell;* i. e. a closed vesicle, or minute bag, generally imperceptible to the naked eye, its form and characters only determinable by microscopic examination.

Every organised being, in fact, is made up of an aggregation of an infinite number of *cel's;* and by studying carefully all the tissues, they may generally be resolved into a primordial cellular arrangement, the cells having experienced, by pressure, coalescence, and other causes, various modifications in their appearance and connections. Here is a wide field of intricate research, in which we cannot but suspect the imaginations of microscopical observers to have supplied some of the data for their deductions. The details we could not of course, if we were competent, here enter upon. Each *cell* which enters into the organism, in a certain sense, possesses an independent vitality—it is a distinct individual, and is endowed with the faculty of growing from its own inherent properties, and of reproducing itself by the production of germs, (called *nuclei* and *nucleoli*) which gradually become developed, and take the place of the parent cell. And this law of succession is as uniform and invariable with reference to these imperceptible corpuscles, as it is with reference to the entire organism which they collectively compose. These are the general principles of the production of the solid structures; but the cell-theory extends much farther than this. It attributes to the prosence and active agency of cells nearly all the functions of the living organism. Absorption, secretion, respiration, nutrition, assimilation, all are accomplished by these minute corpuscles. For these several and widely different processes they not only exist in every part, but according to their location, and the results to be attained, they are possessed of peculiar vital endowments. For example, cells situated at the extremities of the Villi of the small intestines possess endowments qualifying them to draw in nutritious material, discharge their contents into the lacteals, when their existence terminates, and their places are supplied by other cells developed from germs, or nuclei, which were contained within the former. Again, epithelial cells on the surface of mucus membrane, and in the mucus follicles, possess the faculty of separating from the blood and combining the elements of mucus, and, in the stomach, the gastric juice, with which becoming distended the cell walls burst or liquefy, and the secreted fluids are poured into the digestive canal, the active agents of the process, at the same time, their mission having been accomplished, giving place to others by which the same process is to be repeated. Every gland secretes, thus, its peculiar fluid by means of Epithelial cells successively developing themselves, and fulfilling the objects of their temporary being, upon the membrane lining the imperceptible tubes or follicles into which these organs by a last anatomical analysis are resolvable. So, each function is performed through the instrumentality of cells quite uniform in appearance and structure, but widely diverse in vital endowments. Not only are these cells present and active in connection with the solids, but they constitute the organization of the fluids—the blood, lymph and chyle. Here they possess properties and offices as distinct and important to life, as in the case of the solids. Finally, reproduction of the species, in like manner, conforms to the general plan. The secretion of the male sperm is effected, like other secretions, by means of cells. The spermatozoa, formerly erroneously supposed to be animalcules, are germs, or nuclei, which, when placed under the appropriate conditions, become embryos of the future being.

To the reader to whom these views are novel, this slight sketch may perhaps serve to excite curiosity, and prompt farther enquiries. Enough has been positively determined to render it incumbent upon the profession to examine into their merits. Taken collectively that they constitute a theory only, will probably be acknowledged by most Physiologists. On this subject, however, Dr. Carpenter takes a bold position. He believes that "the inferences which may be drawn from the observations on this subject, that have rapidly accumulated during the last few years, are entitled to hold the same rank in Physiological Science as that taken by the doctrine of mutual attraction in General Physics, or of Elective Affinity in Chemistry."—(*Preface.*)

We are greatly disposed to doubt if the amount of actual knowledge in the present condition of the science authorises this assumption.

One consideration is to be borne in mind in connection with this subject: although, assuming the theory of cells to be substantially true, it carries us much farther into the arcana of vital operations than we had previously been able to penetrate, still, the horizon which limits our view is really the same as before. Whence the faculty of each tissue to assume its peculiar characteristics, and of each organ to develop and renew itself after a determinate size, and type of conformation? Whence the vital endowments of cells by which the different functions are performed with such precision and invariable uniformity? These questions remain unanswered. We have, indeed, scarcely advanced a step toward their solution, even by instituting new ultimate principles: for what does the study of cells teach us except historical facts respecting the conditions of organization under which the vital principles operate? They throw no new light on these vital principles, by enabling us to affiliate them with others already known, or to establish among themselves new generalizations.

There is another feature of Dr. Carpenter's work upon which we will offer a few comments. We refer to his views respecting the position which properly belongs to *vitality*, as expressive of a single force, or of an assemblage of forces or principles, in Physiological science. Here we have one of the most vexed of questions in the present stage of Physiological progress. Opinions are sufficiently divided, and enough feeling commingled with the subject, to give rise occasionally to some acrimony of discussion by the advocates of different doctrines. Some of the *ultra vitalists* appear to

)e extremely jealous of the threatened encroachments of chemical ınd physical science upon the domain, which, in their view, belongs exclusively to *life*. They seem to regard a factitious essence so entitled, or a collection of forces called *vital properties*, as peculiarly inviolable, and efforts to explain the phenomena attributed to this essence, or to these properties, by reference to the same laws which preside over inorganic nature, are deemed more than futile—in fact, a kind of sacrilege. On the other hand, the astonishing impulse which of late the science of chemistry has received, particularly in its relations to the phenomena of the organised creation, has doubtless led some minds to extend its application in this direction too far. The attempt to supersede entirely forces peculiar to life by resolving all vital phenomena into chemical laws, is an *ultraism* of the *chemical school*, not less illogical and pernicious than the opposite extreme. In all such instances, truth, of necessity, lies somewhere between. And, in our opinion, Dr. Carpenter takes the ground most in accordance with a truly philosophical view of the subject. It is hardly necessary to say that he discards the supposition of a kind of vicegerency exercised by an imaginary sentient, rational entity called the *vis vitæ* or *vital principle*, or *force*. We can hardly realise that so illogical and unwarrantable an assumption is seriously entertained by many Physiologists ; yet we are well aware that it enters metaphorically to a great extent in common parlance. That in this way it may have, to a considerable extent, influenced our thoughts and reasoning, and thereby been a source of evil, is highly probable. We are glad, therefore, that Dr. C. deems it sufficiently important to adduce the evidences of its absurdity. On this subject a late number of the New Orleans Medical Journal contains a very interesting and able paper by Dr. Harrison, one of the editors of that journal.

This doctrine is very different from that which recognises certain *vital forces*, or *principles*, as ultimate facts of generalization. The former is a gratuitous assumption, the latter results of logical inductions. Dr. Carpenter by no means repudiates such principles, but illustrates, in a very clear and satisfactory manner, the existence of *contractility*, for an example, as a distinct, peculiar vital force, being ›s independent of the forces in inorganic nature, as the latter are distinct from each other. In the present condition of science, the existence of these principles must be acknowledged; but we do not conceive that any especial deference is due to them from their connection with organization, making it heresy to refuse to acknowledge them. What if the chemical philosopher should succeed in embracing all the phenomena of life within the principles and laws of his favorite science, where is the sacrilege? Are not the forces which regulate the actions of inorganic atoms as inscrutable in their nature, as wonderful in their character, and as emphatically expressions of the Divine Will, as the vital forces? Is it for human philosophy to institute gradations of rank among the phenomena of nature, and designate a certain class as deserving peculiar respect, when all are alike mysterious and incomprehensible, except in some of their wise and beneficent results? Certainly this involves infinitely greater presumption than the attempt to effect a coalescence of vital and physical laws, although the latter is frequently stigmatised with this appellation. For ourselves, we have no apprehension of the inroads of chemistry or physics on the provinces of physiology. While we much doubt if the distinctive character of the now vital principles will ever be removed by the extension of chemical or physical laws, yet we would say to those who are disposed to prosecute the endeavor by legitimate methods of logic, God speed the undertaking! It is truth that we require, and should seek for, and human experience, as well as reason, suffices to show that the criteria of new truth are not to be derived exclusively from past or present doctrines, still less from our prejudices, however deeply rooted, or sanctified by time.

Our readers will perceive that instead of making Dr. Carpenter's work the subject of a formal review, we have merely indulged some discursive remarks on some of the topics which the work embraces. Considering the elementary character of the work, and its claims upon the student and the practitioner, we should incur the risk of doing injustice to our readers, as well as to the author, by an attempt to give a critical analysis of its contents. The work itself is prepared with as much abreviation as the nature and importance of the subjects will permit; and we should certainly feel that our labors were unprofitable if we were to do ought to supersede or diminish the necessity, in any case, of bestowing upon the work itself a careful perusal.

We commend the work, not alone from its intrinsic interest and value, but from a firm conviction that keeping pace with the progress of physiological knowledge is the most effective method of advancing and promoting the progress of pathology and practical medicine. We regard the facts and principles of physiology as the point of departure for the determination deductively of the larger proportion of rational pathological principles ; and that, other things being equal, he who is most thoroughly conversant with physiological science will be the most philosophical and successful practitioner. By this remark, we do not mean to disparage the importance of knowledge derived from the observation and comparison of pathological phenomena independently of the healthy organism. The latter are by no means to be neglected. But if medicine will ever have claims to be considered a rational art, it is clear that its rationality will consist primarily and chiefly in the ability to trace with precision and particularity all the circumstances connected with the abnormal deviations from healthy actions. Hence, it follows, as an equally clear inference, that just in proportion to our actual knowledge of the normal actions going on in the organism shall we acquire the ability to study and determine the laws and conditions which regulate, on the one hand, the variations which constitute disease, and, on the other hand, the processes by which healthy physiological actions are restored. We had designed, before penning these remarks, to have considered this topic more fully, and in connection with it, to have advocated the propriety of giving to physiology a more important place among the departments of medical study than is usually assigned to it. The length, however, to which our article has already extended renders it necessary to await a future occasion.

